# The CRCbiome study: a large prospective cohort study examining the role of lifestyle and the gut microbiome in colorectal cancer screening participants

**DOI:** 10.1186/s12885-021-08640-8

**Published:** 2021-08-18

**Authors:** Ane Sørlie Kværner, Einar Birkeland, Cecilie Bucher-Johannessen, Elina Vinberg, Jan Inge Nordby, Harri Kangas, Vahid Bemanian, Pekka Ellonen, Edoardo Botteri, Erik Natvig, Torbjørn Rognes, Eivind Hovig, Robert Lyle, Ole Herman Ambur, Willem M. de Vos, Scott Bultman, Anette Hjartåker, Rikard Landberg, Mingyang Song, Hege Salvesen Blix, Giske Ursin, Kristin Ranheim Randel, Thomas de Lange, Geir Hoff, Øyvind Holme, Paula Berstad, Trine B. Rounge

**Affiliations:** 1 Section for Colorectal Cancer Screening, Cancer Registry of Norway, Oslo, Norway; 2 Department of Research, Cancer Registry of Norway, Oslo, Norway; 3 Department of Tumor Biology, Institute for Cancer Research, The Norwegian Radium Hospital, Oslo University Hospital, Oslo, Norway; 4 Department of Medical Biochemistry, Oslo University Hospital, Oslo, Norway; 5 Institute for Molecular Medicine Finland, HiLIFE, University of Helsinki, Helsinki, Finland; 6 Department of Multidisciplinary Laboratory Science and Medical Biochemistry, Genetic Unit, Akershus University Hospital, Lørenskog, Norway; 7 Department of Informatics, Centre for Bioinformatics, University of Oslo, Oslo, Norway; 8 Department of Microbiology, Oslo University Hospital, Oslo, Norway; 9 Department of Medical Genetics, Oslo University Hospital and University of Oslo, Oslo, Norway; 10 Centre for Fertility and Health, Norwegian Institute of Public Health, Oslo, Norway; 11 Department of Microbiology and Infection Control, Akershus University Hospital, Lørenskog, Norway; 12 Department of Natural Sciences and Health, Oslo Metropolitan University, Oslo, Norway; 13 Laboratory of Microbiology, Wageningen University, Wageningen, The Netherlands; 14 Human Microbiome Research Program, Faculty of Medicine, University of Helsinki, Helsinki, Finland; 15 Department of Genetics and Lineberger Comprehensive Cancer Center, University of North Carolina, Chapel Hill, NC USA; 16 Department of Nutrition, University of Oslo, Oslo, Norway; 17 Department of Biology and Biological Engineering, Division of Food and Nutrition Science, Chalmers University of Technology, Gothenburg, Sweden; 18 Department of Epidemiology, Harvard T.H. Chan School of Public Health, Boston, MA USA; 19 Department of Nutrition, Harvard T.H. Chan School of Public Health, Boston, MA USA; 20 Clinical and Translational Epidemiology Unit and Division of Gastroenterology, Massachusetts General Hospital and Harvard Medical School, Boston, MA USA; 21 Department of Drug Statistics, Norwegian Institute of Public Health, Oslo, Norway; 22 School of Pharmacy, University of Oslo, Oslo, Norway; 23 Cancer Registry of Norway, Oslo, Norway; 24 Medical Department, Sahlgrenska University Hospital-Mölndal, Mölndal, Sweden; 25 Department of Molecular and Clinical Medicine, Sahlgrenska Academy, University of Gothenburg, Gothenburg, Sweden; 26 Department of Medical Research, Bærum Hospital, Bærum, Norway; 27 Department of Research, Telemark Hospital, Skien, Norway; 28 Department of Medicine, Sorlandet Hospital Kristiansand, Kristiansand, Norway; 29 Institute for Health and Society, University of Oslo, Oslo, Norway

**Keywords:** Diet, Lifestyle, Prescription drugs, Gut microbiome, Metagenomics sequencing, Biomarkers, Screening, FIT, iFOBT, Colonoscopy, Adenoma, Colorectal cancer, Advanced neoplasia

## Abstract

**Background:**

Colorectal cancer (CRC) screening reduces CRC incidence and mortality. However, current screening methods are either hampered by invasiveness or suboptimal performance, limiting their effectiveness as primary screening methods. To aid in the development of a non-invasive screening test with improved sensitivity and specificity, we have initiated a prospective biomarker study (CRCbiome), nested within a large randomized CRC screening trial in Norway. We aim to develop a microbiome-based classification algorithm to identify advanced colorectal lesions in screening participants testing positive for an immunochemical fecal occult blood test (FIT). We will also examine interactions with host factors, diet, lifestyle and prescription drugs. The prospective nature of the study also enables the analysis of changes in the gut microbiome following the removal of precancerous lesions.

**Methods:**

The CRCbiome study recruits participants enrolled in the Bowel Cancer Screening in Norway (BCSN) study, a randomized trial initiated in 2012 comparing once-only sigmoidoscopy to repeated biennial FIT, where women and men aged 50–74 years at study entry are invited to participate. Since 2017, participants randomized to FIT screening with a positive test result have been invited to join the CRCbiome study. Self-reported diet, lifestyle and demographic data are collected prior to colonoscopy after the positive FIT-test (baseline). Screening data, including colonoscopy findings are obtained from the BCSN database. Fecal samples for gut microbiome analyses are collected both before and 2 and 12 months after colonoscopy. Samples are analyzed using metagenome sequencing, with taxonomy profiles, and gene and pathway content as primary measures. CRCbiome data will also be linked to national registries to obtain information on prescription histories and cancer relevant outcomes occurring during the 10 year follow-up period.

**Discussion:**

The CRCbiome study will increase our understanding of how the gut microbiome, in combination with lifestyle and environmental factors, influences the early stages of colorectal carcinogenesis. This knowledge will be crucial to develop microbiome-based screening tools for CRC. By evaluating biomarker performance in a screening setting, using samples from the target population, the generalizability of the findings to future screening cohorts is likely to be high.

**Trial registration:**

ClinicalTrials.gov Identifier: NCT01538550.

**Supplementary Information:**

The online version contains supplementary material available at 10.1186/s12885-021-08640-8.

## Background

Colorectal cancer (CRC) is a major global health burden, accounting for nearly 10% of all cancers diagnosed and cancer-related deaths each year [1]. Although a decline in the age-standardized mortality rate has been observed over the past two to three decades in many countries [2–4], death rates remain high, particularly when diagnosed at later stages (5-year survival rate of 13% for metastatic disease compared to 90% when diagnosed at a localized stage) [1, 5]. The significant contribution to global cancer deaths, together with the worrying rise in incidence rates seen globally [3], especially the recent increase among younger age groups [6, 7], highlights the need for widespread prevention strategies that are both effective and feasible on a large-scale basis.

There are two major precursor lesions of CRC: adenomatous polyps, accounting for the majority of cases, and serrated lesions, estimated to underlie up to 30% of CRC [8]. The progression of precursor lesions to CRC is a long-term process, spanning a period of 10–15 years for most lesions [9]. During this long latency period, most cancers develop asymptomatically, making them difficult to detect at a preclinical stage. Therefore, international guidelines recommend screening, with the aim of detection and removal of precancerous lesions to prevent cancer from occurring, or to detect cancer at the earliest stage possible [10–13].

Screening has been shown to reduce both CRC incidence [14–17] and mortality [14–21] in randomized controlled trials, even though current screening methods have known limitations [22]. At present, the most commonly used screening method is the fecal immunochemical test (FIT) for occult blood, having mostly replaced the less sensitive guaiac-based fecal occult blood test (gFOBT) [23]. Despite being more sensitive, performance characteristics are still suboptimal with regards to sensitivity and specificity, resulting in both missed neoplasms and unnecessary colonoscopy referrals [22]. Of particular concern has been the limited performance in detecting precancerous lesions, representing a missed opportunity given the great potential for cancer prevention following removal of these lesions. There is also evidence that current screening methods perform worse for right-sided tumors, compared to left-sided ones [24], as well as in women compared to men [25, 26]. Thus, there is a requirement for screening methods and tools with improved performance for the entire screening population.

Both observational and experimental evidence point to an important role of the gut microbiome in development and progression of CRC [27]. Numerous studies have demonstrated differences in the gut microbiome of tumor and adjacent non-tumor tissue [28, 29], as well as in stool samples from CRC patients and healthy controls [30–38]. Typically, the presence of a colorectal tumor has been associated with enrichment of pathogenic bacterial species, such as *F. nucleatum*, *E. coli* and *B. fragilis*, and depletion of potentially protective bacteria (e.g. producers of short chain fatty acids (SCFAs)) [27]. Although less studied, there are reports indicating that subjects with precancerous lesions display shifts in their microbial profiles [30, 33, 39], suggesting the presence of microbial changes at early stages of colorectal carcinogenesis.

The gut microbiome is heavily influenced by the environment [40]. Established risk factors for CRC, such as excess body weight, physical inactivity and a Western dietary pattern (typically high in red and processed meat and low in whole grains and dietary fiber) and protective factors, such as dairy products and use of certain medications (e.g. aspirin/NSAIDs and metformin) are suggested to modify the gut microbiome [41]. At the same time, accumulating evidence indicates that modifications of the gut microbiome may allow environmental risk factors to induce malignant transformation [42, 43]. This highlights the complex relationship between the environment and the microbiome in the etiology of CRC.

The connection between a potentially pathogenic gut microbiome and CRC has resulted in a growing interest in the use of gut microbial biomarkers as screening tests for early detection of precancerous and cancerous lesions. Several studies have shown that combining microbiome data with the results of established screening methods, such as gFOBT or FIT, substantially increase the ability to classify groups of individuals with healthy colons, adenoma and CRC [30, 33, 34]. Two recent meta-analyses of metagenome data showed that both taxonomic and functional gut microbial profiles predicted CRC at time of diagnosis with high accuracy [44, 45].

Although results from previous biomarker studies are promising, no microbial biomarkers are currently used in national screening programs. In order to advance the utility of the gut microbiome in screening, additional data from prospective studies are needed.

### Objectives

The primary aim of the CRCbiome study is to develop a classification algorithm for identification of advanced colorectal lesions based on the screened individuals’ gut metagenome, demographics and lifestyle. Secondary aims are to provide a deeper understanding of how the gut microbiome evolves prior to a cancer diagnosis, as well as its interactions with host, lifestyle and environmental factors:

I. Identification of associations of the gut microbiome with advanced colorectal lesions, defined as presence of advanced adenomas, advanced serrated lesions or CRC, at baseline
II. Examination of interactions of the gut microbiome with host factors, diet, lifestyle and medication use on risk of advanced colorectal lesions at baseline
III. Description of changes in the gut microbiome following removal of precursor lesions of CRC

Long-term outcomes (i.e. incidence and mortality of advanced colorectal lesions) will be examined by means of passive follow-up using data from the national registries. The outcome assessment will be aligned with the 10 year follow-up of the Bowel Cancer Screening in Norway (BCSN) trial [46], from which the CRCbiome study recruits participants.

## Methods

### Study design

The CRCbiome study is a prospective cohort study nested within the BCSN trial, which is a pilot for a national screening program, organized by the Cancer Registry of Norway. The BCSN study is designed as a randomized trial comparing once-only sigmoidoscopy with FIT tests every two years for a maximum of four rounds [46]. The trial was started in 2012, with follow-up FIT rounds scheduled to be completed in 2024. Participants randomized to the FIT group who test positive (i.e. hemoglobin > 15 mcg/g feces), are referred for follow-up colonoscopy at their local screening center. Neoplastic lesions detected as part of the screening examination are removed during colonoscopy or elective surgery, if necessary. Biennial FIT testing is discontinued for those having undergone colonoscopy following a positive FIT test.

The CRCbiome study recruits participants from the BCSN trial who receive a positive FIT test. FIT positive participants are selected since they are referred to follow-up colonoscopies in line with the BCSN study protocol and will have detailed clinicopathological information. Conversely, as no diagnostic information is available for those with a negative FIT test, these are not included in the CRCbiome study. Of note, as recruitment for the CRCbiome study started five years after commencement of the BCSN trial, those with positive FIT findings in the first and initial part of the second round of screening in the BCSN were not invited. Even so, due to incomplete participation in the first round of FIT testing, 10% of the CRCbiome participants had their inclusion sample as their first screening test.

Participants are invited to the CRCbiome study prior to their colonoscopy examination. The invitation includes an information letter and two questionnaires (further details given below). FIT-positive fecal samples from the BCSN are retrieved following enrolment and represent the baseline sample of the CRCbiome study. Participants are thereafter contacted 2 and 12 months after colonoscopy for collection of follow-up fecal samples using the same sampling method. Fecal samples are processed for microbiome analysis as they become available to the project.

Based on the colonoscopy examination, participants are categorized into diagnostic groups ranging from no pathological findings to presence of advanced lesions and CRC. The groups selected for analyses will vary depending on aim (see Outcome variables for a complete description of outcomes).

**Fig. 1:**
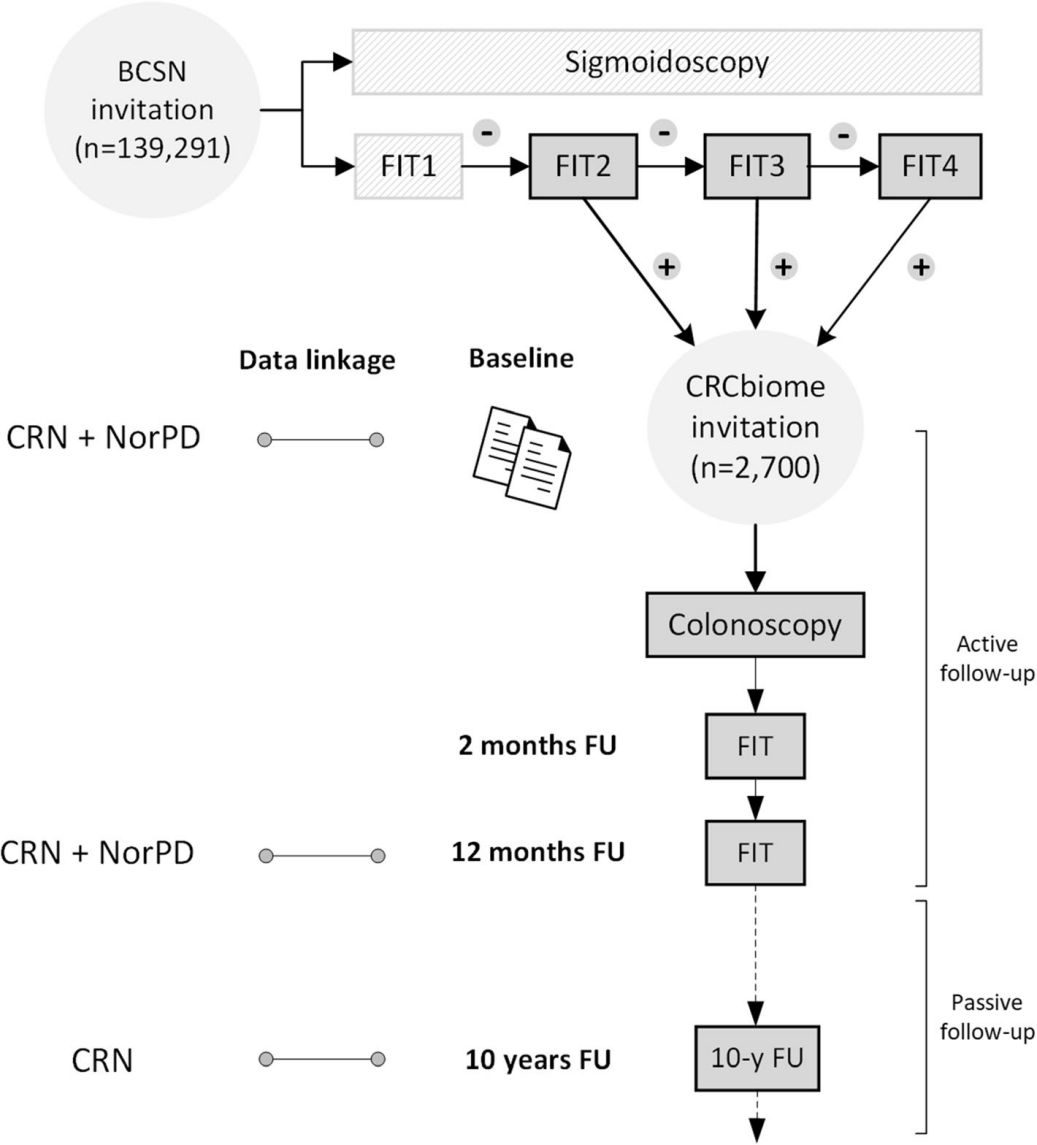
Flowchart of the CRCbiome study, nested within the BCSN. Abbreviations: BCSN, Bowel Cancer Screening in Norway; CRN, Cancer Registry of Norway; FIT, fecal immunochemical test; FU, follow-up; NorPD, Norwegian Prescription Database

Data collected in the CRCbiome study will be linked to national registries, including the Norwegian Prescription Database [47] and the Cancer Registry of Norway [48]. An overview of the study design is shown in Fig. 1. The design and handling of data in the CRCbiome study is in accordance with the STROBE guidelines for observational and metagenomics studies [49–51].

### Participants and eligibility

The BCSN trial includes 139,291 women and men aged 50–74 years in 2012, living in South-East Norway. Of these, 70,096 have been randomized to FIT screening. So far, the cumulative participation rate for the first three FIT rounds has been 68% [46]. All screening participants with a positive FIT test are eligible for the CRCbiome study. Recruitment for the CRCbiome study started in 2017, and will continue until a minimum of 2700 participants have been invited. So far, 2426 have been invited and 1413 (58%) have agreed to participate. With the current participation rate, we expect recruitment to be completed by March 2021 with a final number of participants of about 1600 (see below for the sample size considerations). Recruitment bias will be evaluated by comparing key characteristics of the included participants, such as age, sex and BMI, with those of the BCSN.

The main inclusion and exclusion criteria for the BCSN trial and the CRCbiome study are listed in Table 1.

**Table 1:** Inclusion and exclusion criteria in the BCSN trial and CRCbiome study

Inclusion criteria	
*BCSN*	Aged 50–74 years old in 2012
	Resident in selected municipalities in South-East Norway (Østfold, parts of Akershus and parts of Buskerud)
*CRCbiome*	FIT positive test (i.e. hemoglobin > 15 mcg/g feces) and invited to a follow-up colonoscopy
**Exclusion criteria**	
*BCSN*^a^	Death
	Moving out of the area
	Reaching the upper age limit
	Diagnosed with CRC
*CRCbiome*	Not attending screening colonoscopy
	Low DNA concentration
	Low sequencing yield (< 2 gigabases)

^*a*^*Exclusion criteria apply for individuals who died, moved out of the area, reached the upper age limit, or were diagnosed with CRC before they were due for invitation*

### Recruitment of participants

Eligible subjects are invited after being informed about their positive FIT test and a colonoscopy appointment has been scheduled. Invitations to the CRCbiome study, including the two questionnaires, are sent out by mail a minimum of four days prior to the colonoscopy. Returning at least one of the two questionnaires is regarded as a consent to the study, and includes permission to collect, analyze and store fecal samples, and to retrieve information from questionnaires and health registries.

Both the BCSN trial and the CRCbiome study have been approved by the Regional Committee for Medical Research Ethics in South East Norway (Approval no.: 2011/1272 and 63,148, respectively). The BCSN is also registered at clinicaltrials.gov (Clinical Trial (NCT) no.: 01538550).

### Outcome variables

For the first two aims, the outcome variable will be defined based on the colonoscopy result. Participants will be grouped into four main categories: no confirmed neoplastic findings (Group 1); non-advanced lesions (Group 2); advanced lesions (Group 3); and CRC (Group 4) (Table 2). The advanced lesions group consists of both advanced adenomas (any adenoma with villous histology, high-grade dysplasia or polyp size greater than or equal to 10 mm) and advanced serrated lesions (any serrated lesion with size ≥10 mm or dysplasia). In addition to separating by stage of the carcinogenic process, we may further subdivide lesions by clinicopathological features, including histopathological subtype (e.g. adenomas versus serrated lesions) and site of occurrence (proximal versus distal colon). Also of interest is the potential for distinct roles of environmental factors and the gut microbiome in the two main pathways of colorectal carcinogenesis: the adenoma-carcinoma pathway, and the serrated carcinoma pathway.

**Table 2:** Main outcomes of the screening colonoscopy among CRCbiome participants with preliminary distribution in percentages as of November 2020

Colonoscopy result	Percentages^a^
FIT+, no colonoscopy	3.6
**Group 1**	
Negative	11.2
Polyp without histology^b^	2.4
Non neoplastic findings	18.2
**Group 2**	
Non-advanced serrated lesions^c^	6.4
Non-advanced adenomas (< 3)	23.6
Non-advanced adenomas (≥3)	8.4
**Group 3**	
Advanced serrated lesions^d^	4.4
Advanced adenoma^e^	18.1
**Group 4**	
CRC^f^	3.6

^*a*^*An extended version of this table, with colonoscopy result by FIT round, is shown in* Additional file 1*(Supplementary Table 2). In cases of multiple findings, participants are allocated to the most severe group. Numbers will therefore add up to 100%*

^*b*^*Polyps lost during colonoscopy or where the endoscopist considers biopsy unnescessary, for example hyperplastic polyps in the rectum*

^*c*^*Includes hyperplastic polyps with size < 10 mm and sessile serrated lesions without dysplasia and size < 10 mm*

^*d*^*Defined as any serrated lesions with size ≥ 10 mm or dysplasia*

^*e*^*Defined as any adenoma with either villous histology (≥25% villous components), high-grade dysplasia or polyp size greater than or equal to 10 mm* [52]

^*f*^*Defined as presence of adenocarcinoma arising from the colon or rectum. Collectively, advanced adenoma or CRC are referred to as advanced neoplasia* [52]

For the third aim, the outcome variable will be defined based on the metagenome data. We will monitor several aspects of the gut microbiome to describe the presence of bacterial strains and the functional potential in paired samples during re-establishment of the gut microbiome following bowel cleansing and colonoscopy.

Long-term effects in the study will be assessed 10 years after recruitment is completed. This will include an investigation of incidence and mortality of advanced colorectal lesions.

### Clinical data, biological sampling and questionnaires

#### Assessment of clinical data

As part of the BCSN [46], participants are contacted by a study nurse prior to follow-up colonoscopy, to obtain information on medical history. This includes prior colonoscopies and CT colonographies, comorbidities, drug use, gastrointestinal symptoms, smoking habits, and body weight and height (Table 3). A variety of data are collected in relation to the follow-up colonoscopy, including screening outcomes (i.e. presence and clinicopathological characterization of detected lesions) and characteristics relevant to the endoscopic procedure (Table 3). For all lesions detected; size, location, appearance, technique used for removal and tissue sampling, and completeness of removal, are recorded. Both the medical history data and data collected as part of the follow-up colonoscopy, are entered into a dedicated database by the responsible health care provider. A complete overview of the data collected in the BCSN trial can be found elsewhere [46].

**Table 3:** Data sources and output generated in the CRCbiome study

		Time points^a^			
		Baseline	2 months	12 months	10 years
**Clinical data**					
Medical history	Prior colonoscopies and CT colonographies, comorbidities (3 items), drug use (6 items), gastrointestinal symptoms (9 items), smoking habits (1 item) and body weight and height	x			
Screening specific data	FIT value, endoscopic findings, histopathology and clinical diagnoses, type of procedure and bowel preparation used, degree of bowel cleansing, intubation level, duration of colonoscopy, use of sedation or analgesia, reason for ending the examination, if necessary, and recommended surveillance	x			
**Biological samples**					
Fecal samples	Gut microbiome profile, including taxonomic and functional profiles	x	x	x	
**Questionnaires**					
Lifestyle and demographics (LDQ)	Demographic factors (i.e. national background, marital status, education and occupation), smoking and snus habits (up to 5 questions each), physical activity (hours spent on physical activity of light, moderate and high intensity per week and presence of chronic diseases restricting ability of being physically active), use of regular and cultured milk (two frequency questions), mode of delivery at birth, removal of the appendix, recent use of medications (i.e. antibiotic and antacid usage the last three months), presence of chronic bowel disorders and food intolerances (closed and open format questions) and presence of CRC among first-degree relatives	x			
Diet (FFQ)	Energy intake, intake of macro and micronutrients, frequency and/or amounts of 256 foods and drinks consumed^b^, dietary patterns, including meal pattern, body weight and height	x			
**Registry data**					
Cancer registry	Cancer incidence and mortality, clinicopathological characteristics, information on treatment regimens	x		x	x
Prescriptions database	Complete prescription history since 2004	x		x	

^*a*^*In cases of multiple screening colonoscopies, the time of the 2 and 12 months follow-up visits is defined based on the first and last colonoscopy, respectively*

^*b*^*A complete overview of the food items included in the FFQ is given in* Additional file 1*(Supplementary Table 1)*

#### Biological sampling and gut microbiome analysis

##### FIT sampling and storage

Sampling kits for stool sample collection are mailed to the participants three times during the study period, with the first sample being the positive BCSN FIT sample. No restrictions on diet or medication use are required prior to sampling. Stool is collected using plastic sticks, which collect about 10 mg stool. The stool is then stored in 2 ml of buffer containing HEPES (4-(2-hydroxyethyl)-1-piperazineethanesulfonic acid), BSA (Bovine serum albumin) and sodium azide. Samples are then packed in padded envelopes and returned by mail to a laboratory at Oslo University Hospital for analysis and further storage at − 80 °C. Shipping time is estimated to 3–10 days. Immunochemical testing for blood in feces is performed continuously using the OC-Sensor Diana (Eiken Chemical, Tokyo, Japan) as samples are received at the laboratory.

##### DNA extraction

We have shown that fecal matter collected in the FIT sampling procedure yields comparable microbial diversity and composition to fresh frozen stool samples [53].

Thawed samples are transferred to three 500 ml aliquots from the sampling bottle using a blood sampling needle (Vacuette) perforating the plastic lid. Samples are stored at − 80 °C until further processing.

Extraction of DNA is carried out using the QIAsymphony automated extraction system, using the QIAsymphony DSP Virus/Pathogen Midikit (Qiagen), after an off-board lysis protocol with some modifications. Each sample is lysed with bead-beating: a 500 μl sample aliquot is transferred to a Lysing Matrix E tube (MP Biomedicals) and mixed with 700 μl phosphate-buffered saline (PBS) buffer. The mixture is then shaken at 6.5 m/s for 45 s. After the bead-beating, 800 μl of the sample is mixed with 1055 μl of off-board lysis buffer (proteinase K, ATL buffer, ACL buffer and nuclease-free water) as recommended by Qiagen. The sample is incubated at 68 °C for 15 min for lysis. Nucleic acid purification is performed on the QIAsymphony extraction robot using the Complex800_OBL_CR22796_ID 3489 protocol, a modified version of the Complex800_OBL_V4_DSP protocol. Purified DNA is eluted in 60 μl AVE-buffer (Qiagen). DNA purity is assessed using a Nanodrop2000 (Thermo Fisher Scientific, USA), and the concentration is measured by Qubit (Thermo Fisher Scientific, USA).

##### Metagenome sequencing

Libraries for metagenome sequencing are prepared from extracted DNA at the sequencing laboratory of the Institute for Molecular Medicine Finland FIMM Technology Centre, University of Helsinki (P.O. Box 20, University of Helsinki, Finland) using Illumina sequencing, with the aim of producing 3 gigabases of DNA sequence per sample.

In details, 29 μl of extracted DNA is purified and concentrated by adding an equal volume of AMPure XP (Beckman Coulter Life Sciences, Indianapolis, IN, USA). Purification is then performed as per the manufacturer’s instructions. The purified samples are eluted to 17 μl of 10 mM Tris-HCl, pH 8.5, and DNA concentrations are determined by Quant-iT PicoGreen dsDNA Assay Kit (Thermo Fisher Scientific, Waltham, MA, USA). The samples are normalized to a maximum concentration of 3.3 ng/μl, resulting in DNA inputs of 25 ng or less.

Sequencing libraries are prepared according to the Nextera DNA Flex Library Prep Reference Guide (v07) (Illumina, San Diego, CA, USA), with the exception that the reaction volumes are scaled down to ¼ of the protocol volumes. The libraries are amplified according to the protocol with 7 PCR cycles. All the library preparation steps are performed on a Microlab STARlet (Hamilton Company, Reno, NV, USA) and Biomek NX^P^ (Beckman Coulter Life Sciences, Indianapolis, IN, USA) liquid handlers running custom scripts.

DNA concentrations of the finished libraries are determined with Quant-iT PicoGreen dsDNA Assay. Libraries are combined into pools containing 240 libraries with 4.5 ng of each library using Echo 525 Acoustic Liquid Handler (Beckman Coulter Life Sciences, Indianapolis, IN, USA). Library pools are size-selected to a fragment size range between 650 and 900 bp using BluePippin (Sage Science Beverly, MA, USA).

Sequencing is performed with the Illumina NovaSeq system using S4 flow cells with lane divider (Illumina, San Diego, CA, USA). Each pool is sequenced on a single lane. Read length for the paired-end run is 2 × 151 bp.

##### Processing and analysis of sequencing data

Sequencing data are transferred to a platform for secure storage and analysis of sensitive research-related data at the University of Oslo [54]. The analysis of metagenomic sequencing data is handled in a uniform manner using a customizable workflow manager [55]. To establish a quality-filtered dataset, standard filters are applied: sequences corresponding to adapters used in library preparation, being of low quality [56] and those mapping to the human genome [57], with subsequent quality control of filtered sequencing reads [58].

Taxonomic classification and determination of microbial gene content, including functional annotation (e.g. using gene ontology and KEGG databases) will be performed using publicly available tools. Abundance measures will be used to calculate taxonomic and functional alpha and beta diversity, as well as serving as input for machine learning approaches aimed at producing classifiers for high-risk individuals in a data-driven manner. Further metagenome-derived measures may include identification of metagenome-assembled genomes, strain-level analysis and description of the gut virome.

#### Questionnaires

Two questionnaires are used to collect data on diet, lifestyle and demographic information; a food frequency questionnaire (FFQ) and a general lifestyle and demographics questionnaire (LDQ). Self-reported dates of questionnaire completion are registered in the project database. Returned questionnaires are reviewed manually before scanning and further processing. In cases of low-quality data, participants are contacted for clarification.

##### Assessment of dietary intake

Dietary intake is assessed using a semiquantitative, 14-page FFQ, designed to assess the habitual diet during the preceding year. The questionnaire is a modified version of an FFQ developed and validated by the Department of Nutrition, University of Oslo [59–64]. The questionnaire has been validated for both energy intake [59–61], intake of macro and micronutrients [59, 61, 64], as well as selected food items and groups [61–64]. The questionnaire includes 23 main questions, covering a total of 256 food items, as well as a free-text field for entries of food items not covered by the questionnaire. For each food item (except one on preferred types of fat for cooking), participants are asked to record frequency of consumption, ranging from never/seldom to several times a day, and/or amount, typically as portion size given in various household units (e.g. deciliters, glasses, cups, spoons). In total, there are 249 questions on frequency, 204 on portion size, one on preferences and nine other, mostly related to meal patterns (Additional file 1**, supplementary Table 1**).

As with any dietary assessment method, the FFQ is prone to errors due to inaccurate reporting and missing answers. Therefore, to mitigate such errors, a standardized framework for how to review and evaluate FFQ quality has been developed. A detailed overview of the framework is given in Additinoal file 2**, supplementary Fig. 1**. In brief, incoming FFQs are reviewed by trained personnel according to a set of predefined criteria. Scanning of questionnaires is performed using the Cardiff TeleForm program (Datascan, Oslo, Norway). The dietary calculation system KBS (short for “**K**ost**b**eregnings**s**ystem”), developed at the Department of Nutrition, University of Oslo, is used to calculate food and nutrient intake. The latest version of the food database (i.e. AE-18 or newer) will be used, which is largely based on the Norwegian Food Composition Table [65]. In line with common practice in nutrition studies, missing answers are imputed as zero intake [61, 63, 66, 67] and observations with extreme energy intake levels in both the upper and lower range will be excluded [68].

The main focus of the dietary analyses will be on foods and drinks linked to the risk of CRC and its precursor lesions, including intakes of alcohol, red and processed meat, wholegrains, foods containing dietary fiber, dairy products and calcium supplements [69]. Dietary intake will also be studied holistically by employing various dietary indices such as the 2018 World Cancer Research Fund/American Institute for Cancer Research (WCRF/AICR) index for adherence to cancer prevention recommendations [70].

##### Assessment of lifestyle and demographic data

Lifestyle and demographic data are assessed using a four page questionnaire based on questions used in previous national surveys [71, 72]. Prior to the study start, the questionnaire was piloted in a targeted population and adjusted based on feedback from pilot study participants. The questionnaire has ten main questions covering demographic factors (national background, education, occupation and marital status), diagnosis of CRC among first-degree relatives, presence of chronic bowel disorders and food intolerances, removal of the appendix, mode of delivery at birth, smoking and snus (i.e. smokeless tobacco) habits, recent use of medications, the past years’ physical activity level and use of regular and cultured milk, which is not completely covered in the FFQ (see Table 3 for a detailed overview). In the questions concerning smoking and snus habits, participants are asked to recall their current habits, including the daily number of cigarettes/snus portions, as well as years since possible cessation and total years of use. Questionnaires are scanned and processed using the Cardiff TeleForm program (InfoShare, Oslo, Norway).

### Registry data

Data collected in the CRCbiome study will be linked to national registries, including the Norwegian Prescription Database and the Cancer Registry of Norway, using personal identification numbers. Complete data linkages will be undertaken twice during active follow-up: after all participants have completed baseline and diagnostic information from follow-up colonoscopies is available, and then after the one-year follow-up is completed. In addition, linkage to the Cancer Registry of Norway will be performed at least once during the 10 year follow-up period.

#### Norwegian prescription database

The Norwegian Prescription Database [73] will be used to obtain information on medication history prior to CRC screening, and during the first year of follow-up. The registry contains data on all medications prescribed to Norwegian citizens since 2004. Prescription drugs are categorized according to the Anatomical Therapeutic Chemical (ATC) system, a hierarchical classification system developed by the WHO [74, 75]. For each drug, the number of packages dispensed, the number of defined daily doses (DDD), the prescription category, and the date of dispensing are registered.

Linkage to the Norwegian Prescription Database enables an in-depth analysis of associations between drug use, the gut microbiome and advanced colorectal lesions. Initially, we will perform drug-wide association analyses to screen for potential associations, adjusting for key covariates. Detected associations will then be examined in detail, including a more refined categorization of drug variables, robust covariate adjustments as well as the analysis of timing and dose-response relations. Prescription histories will also be used as a proxy for life-long burden of chronic diseases. To examine the representativeness of the drug profiles discoverd in the CRCbiome study, a randomly selected control group drawn from the National Population Registry, might be included.

#### Cancer registry of Norway

Information on clinicopathological characteristics, cancer therapy, as well as outcomes assessed as part of passive follow-up, will be obtained from the Cancer Registry of Norway [76]. The Cancer Registry of Norway has recorded incident cancer cases on a nationwide basis since 1953 and has been shown to have accurate and almost complete ascertainment of cases (98.8% for the registration period 2001–2005) [77]. According to recent estimates, about 93% of all cancer cases and ≥ 95% of cancers in the colon and rectum are morphologically verified [48]. Cancer diagnoses are recorded using the International Classification of Diseases, version 10 (ICD-10). Mortality data in the registry are obtained from the Cause of Death Registry and coded using the same ICD-10 categories as for the incidence data.

### Data processing and management

**Fig. 2:**
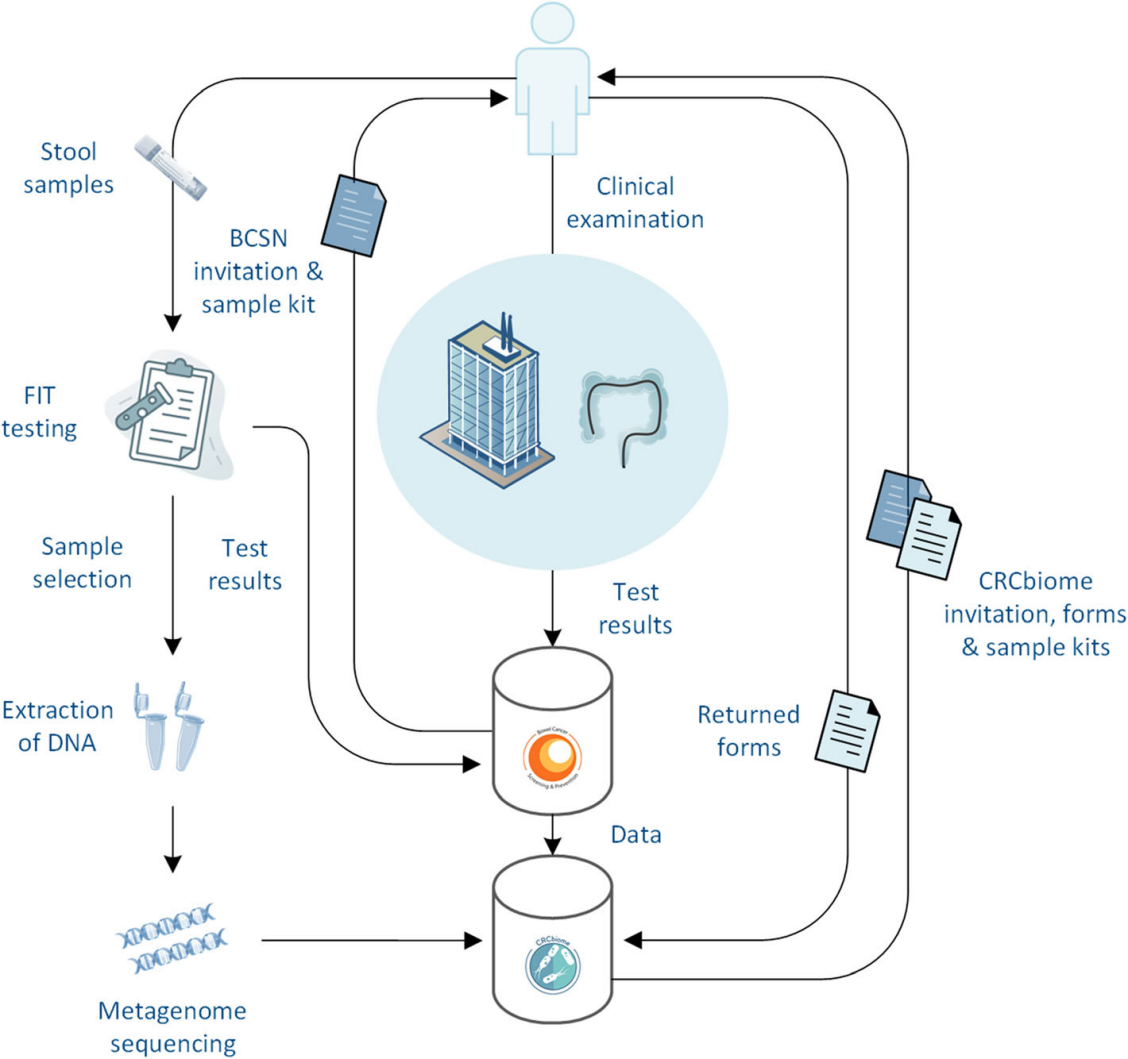
Simplified version of the data generation process in CRCbiome. The figure is created based on free images from Servier Medical Art (Creative Commons Attribution Liscence, creativecommons.org/liscences/by/3.0/) and Stockio (https://www.stockio.com/)

To facilitate project administration, including recruitment and follow-up of participants, custom software has been developed. This application communicates with two project specific databases (i.e. the BCSN and CRCbiome databases). Only authorized data manager personnel have complete access to the datasets. A simplified version of the data generation process is depicted in Fig. 2.

In line with common practice for linkage with national registries [78], linked data will receive unique ID numbers specific to the particular project. Linkage of research data will be performed by the data controller. For the metagenome data, which due to its size cannot be transferred using ordinary methods, linkage will be performed in-house by an independent data manager without access to other parts of the data than those strictly necessary for linkage.

All data collected in the CRCbiome study will be stored and analyzed at a platform for secure handling of sensitive research-related data, operated by the University of Oslo [54]. Access to research data for external investigators, or use outside of the current protocol, will require approval from the Norwegian Regional Committee for Medical and Health Research Ethics and a data access committee (information available on the project web site [79]). Research data are not openly available because of the principles and conditions set out in articles 6 [1] (e) and 9 [2] (j) of the General Data Protection Regulation (GDPR).

### Statistical analyses and sample size considerations

The number of participants to include was chosen with the aim of providing adequate power for the development of a highly sensitive classification algorithm via data-driven analyses of gut metagenomes that will accurately identify FIT-positive individuals in need of clinical intervention.

The classifier will be trained using counts of taxonomic units, signature and genes categorized according to gene ontology or pathway membership from metagenomes, FFQ, demographic and lifestyle data as input variables, and advanced colorectal lesions as outcome (i.e. group group 3 and group 4, Table 2). The CRC risk classification will be done using machine learning algorithms suited to metagenome data, such as lasso regression [80], support-vector machines [81], random forests [82], multi-layer perception neural networks [83] and scalable tree boosting [84] algorithms. Evaluation of the classifier will be conducted in a leave-out test set. As outlined below, we believe that with sufficient sample size, development of a classifier with a sensitivity of 0.95 is achievable in the training set, being within the range of published reports [30, 33].

Interpretation of the classifier will be sought by post hoc analysis, quantifing the importance of individual features (taxa, genes and pathways) in making predictions. Stratified analyses will be done to evaluate the classifier within different subgroups of the population (e.g. by age group, sex and screening center).

With a projected classifier sensitivity of 0.95 and a minimally acceptable sensitivity of 0.8, at 80% power and 95% confidence level, 50 participants with advanced colorectal lesions are required in the test set [85]. Classifier specificity in the setting of FIT-positive individuals will have a lower requirement, and we therefore set the expected classifier specificity to 0.75 and a minimally acceptable specificity of 0.6, thus requiring 100 participants with normal findings in the test set. Based on initial recruitment, we expect a participation rate of 58%, with 26% of participants having findings of advanced lesions or CRC (Table 2). By inviting 2700 FIT-positive BCSN participants, and splitting the training and test sets 80/20, a projected number of 1253 and 313 participants will constitute the training and test sets, respectively, which will include adequate numbers of participants with both advanced colorectal lesions and normal findings in the test set. With this sample size, we will also be able to perform stratified analyses. The machine learning analyses will be complemented by various multivariate regression analyses, stratified by the covariates outlined above.

## Discussion

CRC remains a major public health challenge with substantial personal and societal costs [22]. Screening is an effective measure to reduce disease burden [22]. However, current screening methods suffer from limitations, limiting the number of preventable cases. Innovative use of currently available methods represents a promising avenue for improvements in CRC prevention [22]. The current study is designed to contribute to the development of microbial biomarkers, using metagenome sequencing and comprehensive questionnaire and registry data for improved detection of advanced lesions and CRC in a FIT-positive population. The CRCbiome study is unique in that it uses data from the screening population to develop relevant biomarkers.

The idea of using microbial biomarkers to increase the performance of CRC screening has received increased attention with the adoption of high-throughput characterization of the gut microbiome. Ideally, combining microbial biomarkers with FIT testing could achieve the sensitivity of direct visualization methods and the uptake of non-invasive fecal tests. Several studies have demonstrated improved ability to discriminate individuals with healthy colons from those with advanced neoplasia when adding microbial biomarkers in the prediction model, more so for carcinoma (area under the curve (AUC) of 0.87–0.97 [30, 33, 34]) than adenoma (AUC of 0.76 [33]). Despite great promise, these studies have typically been limited by small sample sizes [30, 32–34], cross-sectional designs [30–34], use of suboptimal or low-resolution methods to study the gut-microbiome [30–33] and lack of data on important confounders [30–34]. The CRCbiome study seeks to address several of these shortcomings.

Major strengths of the CRCbiome study include its large sample size and prospective nature, use of state of the art methodology for studying the gut microbiome and access to detailed information on likely confounders of the relationship between the gut-microbiome and advanced colorectal lesions. A further strength of the study is in its organization and logistics structure, being nested within the BCSN. The immediate availability of clinically verified outcome data, via follow-up colonoscopies and cancer registry data, allow for prospective investigations on multiple outcomes relevant to the screening population (e.g. polyp recurrence). Access to comprehensive high-quality data on diet and lifestyle, including complete prescription histories, also enables the investigation of the predictive performance of more broad classifiers, laying the ground for personalized screening strategies, including risk-stratified approaches.

With a study population solely consisting of FIT positive participants, the projected number of individuals with high-risk lesions or CRC is relatively high (about 409 (26%), group 3 and 4, Table 2), thereby increasing the power to achieve accurate classification of advanced colorectal neoplasms. Still, whether findings in this population extends to cases missed by FIT testing is unknown.

Collection of follow-up samples at 2 and 12-months post colonoscopy represents an extension of the cross-sectional design of most prior studies, shedding light on the development of the gut microbiome following colonoscopy with or without removal of CRC precursor lesions. While there are examples of shifts in microbial profiles following colonoscopy, the gut microbiome typically reverts to the initial state within weeks [86]. Deviations from re-establishment of the gut microbiome both in the medium and long term have the potential for causal interpretations.

The study also has some limitations. Exclusive selection of FIT positive participants may limit the generalizability of the findings to those with bleeding neoplastic lesions. Consequently, improvements in diagnostic performance may be limited to specificity, and thus the ability to correctly classify healthy individuals. However, since lesions tend to bleed intermittently [87] and the study aims to identify potential causal pathways, we consider it likely that the identified biomarker also may have improved sensitivity in the screening population as a whole.

A further limitation is the lack of information on fecal metrics such as the Bristol stool scale, which has been shown to be an important determinant of microbiota richness and variance [88]. However, variation in microbiome profile due to stool consistency could likely be explored by use of gastrointestinal symptoms as a surrogate, data on which is available in the BCSN database.

Lastly, lack of follow-up data on diet and lifestyle may complicate the interpretation of microbial changes following colonoscopy. Even though prior studies in comparable study populations show that potential changes in diet and lifestyle following screening are modest [89, 90], caution in interpretation of follow-up samples is warranted.

The CRCbiome study represents a valuable source of data for further research. An example is access to complete prescription histories from the Norwegian Prescription Database that enables in-depth analyses of associations between a broad range of medications, microbial features and neoplasia risk, both during short and long-term follow-up. The fecal samples collected are also biobanked and can be used for other purposes beside the study aims of the current protocol. For instance, in addition to metagenome sequencing, the fecal samples can potentially be used for other omics analyses, such as transcriptome and metabolome analysis. All tissue specimens removed during colonoscopy are also available to the project, enabling in-depth molecular profiling.

The integration of a microbiome-based biomarker into national CRC screening programs is a long-term process, requiring many steps before enabling full implemtation. Ideally, the discovery phase will lead to the identification of a few selected features that will predict the occcurence of advanced colorectal lesions with high accuracy. These could then be combined by means of a biomarker panel for the development of a rapid test, which, following rigorous validation and testing, has the potential of being integrated into screening programs. The cost-effectivness of adding a microbial biomarker to the FIT test should be carefully evaluated before implementation.

## Conclusion

The CRCbiome study investigates the role of the gut microbiome, and its interactions with host factors, diet and lifestyle, in early stage colorectal carcinogenesis. Information obtained from this project will guide the development of a microbial biomarker for accurate detection of advanced colorectal lesions. By performing biomarker discovery within a screening population, the generalizability of the findings to future screening cohorts is likely to be high.

## Supplementary Information

**Additional file 1.** Contains two supplementary tables.

**Additional file 2.** Contains a supplementary figure with figure title and legend.

**Additional file 3.** Contains the STROBE checklist for observational studies.

## Data Availability

Due to the principles and conditions set out in articles 6 [1] (e) and 9 [2] (j) of the General Data Protection Regulation (GDPR), research data generated in the CRCbiome study are not openly available. Further information on access to CRCbiome data can be found on the project web site [79]).

## Abbreviations

AICR: American Institute for Cancer Research
ATC: Anatomical Therapeutic Chemical
AUC: area under the curve
BCA: bovine serum albumin
BCSN: Bowel Cancer Screening in Norway
Bp: base pair
CRC: colorectal cancer
CRN: Cancer Registry of Norway
CT: computed tomography
DDD: defined daily doses
DNA: deoxyribonucleic acid
DPIA: Data Processing Impact Assessment
FFQ: food frequency questionnaire
FIMM: Institute for Molecular Medicine Finland
FIT: fecal immunochemical test
FU: follow-up
gFOBT: guaiac-based fecal occult blood test
ICD: International Classification of Diseases
KBS: Kostberegningssystem (“Dietary calculation system”)
KEGG: Kyoto Encyclopedia of Genes and Genomes
LDQ: lifestyle and demographic questionnaire
NCT: National Clinical Trial
NorPD: Norwegian Prescription Database
NSAIDs: non-steroid anti-inflammatory drugs
PCR: polymerase chain reaction
PBS: phosphate-buffered saline
SCFA: short chain fatty acid
STROBE: Strengthening the Reporting of Observational Studies in Epidemiology
WCRF: World Cancer Research Fund
WHO: World Health Organization

## References

[1] Bray F, Ferlay J, Soerjomataram I, Siegel RL, Torre LA, Jemal A. Global cancer statistics 2018: GLOBOCAN estimates of incidence and mortality worldwide for 36 cancers in 185 countries. CA Cancer J Clin. 2018;68(6):394–424. 10.3322/caac.21492 30207593

[2] Ouakrim DA, Pizot C, Boniol M, Malvezzi M, Boniol M, Negri E, Bota M, Jenkins MA, Bleiberg H, Autier P (2015). Trends in colorectal cancer mortality in Europe: retrospective analysis of the WHO mortality database. BMJ..

[3] Safiri S, Sepanlou SG, Ikuta KS, Bisignano C, Salimzadeh H, Delavari A, Ansari R, Roshandel G, Merat S, Fitzmaurice C, Force LM, Nixon MR, Abbastabar H, Abegaz KH, Afarideh M, Ahmadi A, Ahmed MB, Akinyemiju T, Alahdab F, Ali R, Alikhani M, Alipour V, Aljunid SM, Almadi MAH, Almasi-Hashiani A, al-Raddadi RM, Alvis-Guzman N, Amini S, Anber NH, Ansari-Moghaddam A, Arabloo J, Arefi Z, Asghari Jafarabadi M, Azadmehr A, Badawi A, Baheiraei N, Bärnighausen TW, Basaleem H, Behzadifar M, Behzadifar M, Belayneh YM, Berhe K, Bhattacharyya K, Biadgo B, Bijani A, Biondi A, Bjørge T, Borzì AM, Bosetti C, Bou-Orm IR, Brenner H, Briko AN, Briko NI, Carreras G, Carvalho F, Castañeda-Orjuela CA, Cerin E, Chiang PPC, Chido-Amajuoyi OG, Daryani A, Davitoiu DV, Demoz GT, Desai R, Dianati nasab M, Eftekhari A, el Sayed I, Elbarazi I, Emamian MH, Endries AY, Esmaeilzadeh F, Esteghamati A, Etemadi A, Farzadfar F, Fernandes E, Fernandes JC, Filip I, Fischer F, Foroutan M, Gad MM, Gallus S, Ghaseni-Kebria F, Ghashghaee A, Gorini G, Hafezi-Nejad N, Haj-Mirzaian A, Haj-Mirzaian A, Hasanpour-Heidari S, Hasanzadeh A, Hassanipour S, Hay SI, Hoang CL, Hostiuc M, Househ M, Ilesanmi OS, Ilic MD, Innos K, Irvani SSN, Islami F, Jaca A, Jafari Balalami N, Jafari delouei N, Jafarinia M, Jahani MA, Jakovljevic M, James SL, Javanbakht M, Jenabi E, Jha RP, Joukar F, Kasaeian A, Kassa TD, Kassaw MW, Kengne AP, Khader YS, Khaksarian M, Khalilov R, Khan EA, Khayamzadeh M, Khazaee-Pool M, Khazaei S, Khosravi Shadmani F, Khubchandani J, Kim D, Kisa A, Kisa S, Kocarnik JM, Komaki H, Kopec JA, Koyanagi A, Kuipers EJ, Kumar V, la Vecchia C, Lami FH, Lopez AD, Lopukhov PD, Lunevicius R, Majeed A, Majidinia M, Manafi A, Manafi N, Manda AL, Mansour-Ghanaei F, Mantovani LG, Mehta D, Meier T, Meles HG, Mendoza W, Mestrovic T, Miazgowski B, Miazgowski T, Mir SM, Mirzaei H, Mohammad KA, Mohammad Gholi Mezerji N, Mohammadian-Hafshejani A, Mohammadoo-Khorasani M, Mohammed S, Mohebi F, Mokdad AH, Monasta L, Moossavi M, Moradi G, Moradpour F, Moradzadeh R, Nahvijou A, Naik G, Najafi F, Nazari J, Negoi I, Nguyen CT, Nguyen TH, Ningrum DNA, Ogbo FA, Olagunju AT, Olagunju TO, Pana A, Pereira DM, Pirestani M, Pourshams A, Poustchi H, Qorbani M, Rabiee M, Rabiee N, Radfar A, Rahmati M, Rajati F, Rawaf DL, Rawaf S, Reiner RC, Renzaho AMN, Rezaei N, Rezapour A, Saad AM, Saadatagah S, Saddik B, Salehi F, Salehi Zahabi S, Salz I, Samy AM, Sanabria J, Santric Milicevic MM, Sarveazad A, Satpathy M, Schneider IJC, Sekerija M, Shaahmadi F, Shabaninejad H, Shamsizadeh M, Sharafi Z, Sharif M, Sharifi A, Sheikhbahaei S, Shirkoohi R, Siddappa Malleshappa SK, Silva DAS, Sisay M, Smarandache CG, Soofi M, Soreide K, Soshnikov S, Starodubov VI, Subart ML, Sullman MJM, Tabarés-Seisdedos R, Taherkhani A, Tesfay B, Topor-Madry R, Traini E, Tran BX, Tran KB, Ullah I, Uthman OA, Vacante M, Vahedian-Azimi A, Valli A, Varavikova E, Vujcic IS, Westerman R, Yazdi-Feyzabadi V, Yisma E, Yu C, Zadnik V, Zahirian Moghadam T, Zaki L, Zandian H, Zhang ZJ, Murray CJL, Naghavi M, Malekzadeh R (2019). The global, regional, and national burden of colorectal cancer and its attributable risk factors in 195 countries and territories, 1990–2017: a systematic analysis for the global burden of disease study 2017. Lancet Gastroenterol Hepatol.

[4] Danckert B, Ferlay J, Engholm G , Hansen HL, Johannesen TB, Khan S, et al. NORDCAN: Cancer Incidence, Mortality, Prevalence and Survival in the Nordic Countries. Version 8.2. 2019.

[5] Howlader N, Noone AM, Krapcho M, Miller D, Brest A, Yu M, et al (eds). SEER Cancer Statistics Review, 1975–2017, National Cancer Institute., Bethesda, MD, https://seer.cancer.gov/csr/1975_2017/, based on November 2019 SEER data submission, posted to the SEER web site, April 2020. 2020.

[6] Sung H, Siegel RL, Rosenberg PS, Jemal A. Emerging cancer trends among young adults in the USA: analysis of a population-based cancer registry. Lancet Public Heal. 2019;4:e137–47. 10.1016/S2468-2667(18)30267-6 30733056

[7] Araghi M, Soerjomataram I, Bardot A, Ferlay J, Cabasag CJ, Morrison DS, de P, Tervonen H, Walsh PM, Bucher O, Engholm G, Jackson C, McClure C, Woods RR, Saint-Jacques N, Morgan E, Ransom D, Thursfield V, Møller B, Leonfellner S, Guren MG, Bray F, Arnold MDP (2019). Changes in colorectal cancer incidence in seven high-income countries: a population-based study. Lancet Gastroenterol Hepatol..

[8] Leggett B, Whitehall V (2010). Role of the serrated pathway in colorectal Cancer pathogenesis. Gastroenterology..

[9] Dekker E, Tanis PJ, Vleugels JLA, Kasi PM, Wallace MB (2019). Colorectal cancer. Lancet (London, England).

[10] Bibbins-Domingo K, Grossman DC, Curry SJ, Davidson KW, Epling JW, García FAR (2016). Screening for colorectal cancer: US preventive services task force recommendation statement. JAMA - J Am Med Assoc..

[11] Segnan N, Patnick J (2010). von Karsa L EC. European guidelines for quality Assurance in Colorectal Cancer Screening and Diagnosis - first edition.

[12] Ebell MH, Thai TN, Royalty KJ (2018). Cancer screening recommendations: an international comparison of high income countries. Public Health Rev.

[13] Sung JJY, Ng SC, Chan FKL, Chiu HM, Kim HS, Matsuda T, Ng SS, Lau JY, Zheng S, Adler S, Reddy N, Yeoh KG, Tsoi KK, Ching JY, Kuipers EJ, Rabeneck L, Young GP, Steele RJ, Lieberman D, Goh KL, Asia Pacific Working Group (2015). An updated Asia Pacific consensus recommendations on colorectal cancer screening. Gut..

[14] Atkin W, Wooldrage K, Parkin DM, Kralj-Hans I, MacRae E, Shah U, et al. Long term effects of once-only flexible sigmoidoscopy screening after 17 years of follow-up: the UK Flexible Sigmoidoscopy Screening randomised controlled trial. Lancet. 2017;389:1299–1311. 10.1016/S0140-6736(17)30396-3 Accessed 8 June 2021. 10.1016/S0140-6736(17)30396-3 PMC6168937 28236467

[15] Schoen R, Pinsky P, Weissfeld J, Yokochi L, Church T, Laiyemo A, Bresalier R, Andriole GL, Buys SS, Crawford ED, Fouad MN, Isaacs C, Johnson CC, Reding DJ, O'Brien B, Carrick DM, Wright P, Riley TL, Purdue MP, Izmirlian G, Kramer BS, Miller AB, Gohagan JK, Prorok PC, Berg CD, PLCO Project Team (2012). Colorectal-Cancer incidence and mortality with screening flexible sigmoidoscopy. N Engl J Med.

[16] Segnan N, Armaroli P, Bonelli L, Risio M, Sciallero S, Zappa M, Andreoni B, Arrigoni A, Bisanti L, Casella C, Crosta C, Falcini F, Ferrero F, Giacomin A, Giuliani O, Santarelli A, Visioli CB, Zanetti R, Atkin WS, Senore C, and the SCORE Working Group (2011). Once-only sigmoidoscopy in colorectal cancer screening: follow-up findings of the italian randomized controlled trial - SCORE. J Natl Cancer Inst.

[17] Holme Ø, Løberg M, Kalager M, Bretthauer M, Hernán MA, Aas E, Eide TJ, Skovlund E, Schneede J, Tveit KM, Hoff G (2014). Effect of flexible sigmoidoscopy screening on colorectal cancer incidence and mortality: a randomized clinical trial. JAMA - J Am Med Assoc.

[18] Mandel J, Bond J, Church T, Snover D, Bradley M, Schuman L, et al. Reducing mortality from colorectal cancer by screening for fecal occult blood. Minnesota Colon Cancer Control Study. N Engl J Med. 1993; 10.1056/NEJM199305133281901 8474513

[19] Kronborg O, Fenger C, Olsen J, Jørgensen OD, Søndergaard O. Randomised study of screening for colorectal cancer with faecal-occult-blood test. Lancet. 1996;348(9040):1467–71. 10.1016/S0140-6736(96)03430-7. . 10.1016/S0140-6736(96)03430-7 8942774

[20] Lindholm E, Brevinge H, Haglind E (2008). Survival benefit in a randomized clinical trial of faecal occult blood screening for colorectal cancer. Br J Surg England.

[21] Hardcastle JD, Chamberlain JO, Robinson MHE, Moss SM, Amar SS, Balfour TW, et al. Randomised controlled trial of faecal-occult-blood screening for colorectal cancer. Lancet. 1996;348(9040):1472–7. 10.1016/S0140-6736(96)03386-7. . 10.1016/S0140-6736(96)03386-7 8942775

[22] Ladabaum U, Dominitz JA, Kahi C, Schoen RE. Strategies for Colorectal Cancer Screening. Gastroenterology [Internet]. Elsevier, Inc; 2020;158:418. 10.1053/j.gastro.2019.06.043. . 10.1053/j.gastro.2019.06.043 31394083

[23] Schreuders EH, Ruco A, Rabeneck L, Schoen RE, Sung JJY, Young GP, Kuipers EJ (2015). Colorectal cancer screening: a global overview of existing programmes. Gut..

[24] Haug U, Kuntz KM, Knudsen AB, Hundt S, Brenner H (2011). Sensitivity of immunochemical faecal occult blood testing for detecting left-vs right-sided colorectal neoplasia. Br J Cancer.

[25] Holme Ø, Løberg M, Kalager M, Bretthauer M, Hernán MA, Aas E, Eide TJ, Skovlund E, Lekven J, Schneede J, Tveit KM, Vatn M, Ursin G, Hoff G, NORCCAP Study Group† (2018). Long-term effectiveness of sigmoidoscopy screening on colorectal cancer incidence and mortality in women and men: a randomized trial. Ann Intern Med.

[26] Brenner H, Qian J, Werner S (2018). Variation of diagnostic performance of fecal immunochemical testing for hemoglobin by sex and age: results from a large screening cohort. Clin Epidemiol.

[27] Tilg H, Adolph TE, Gerner RR, Moschen AR. The Intestinal Microbiota in Colorectal Cancer. Cancer Cell. Elsevier Inc.; 2018;33:954–964. 10.1016/j.ccell.2018.03.004 29657127

[28] Kostic AD, Gevers D, Pedamallu CS, Michaud M, Duke F, Earl AM, Ojesina AI, Jung J, Bass AJ, Tabernero J, Baselga J, Liu C, Shivdasani RA, Ogino S, Birren BW, Huttenhower C, Garrett WS, Meyerson M (2012). Genomic analysis identifies association of Fusobacterium with colorectal carcinoma. Genome Res.

[29] Castellarin M, Warren R, Freeman J, Dreolini L, Krzywinski M, Strauss J (2012). Fusobacterium nucleatum infection is prevalent in human colorectal carcinoma. Genome Res.

[30] Zackular JP, Rogers MAM, Ruffin MT, Schloss PD (2014). The human gut microbiome as a screening tool for colorectal cancer. Cancer Prev Res.

[31] Guo S, Li L, Xu B, Li M, Zeng Q, Xiao H, Xue Y, Wu Y, Wang Y, Liu W, Zhang G (2018). A simple and novel fecal biomarker for colorectal cancer: ratio of Fusobacterium nucleatum to probiotics populations, based on their antagonistic effect. Clin Chem.

[32] Liang Q, Chiu J, Chen Y, Huang Y, Higashimori A, Fang J, Brim H, Ashktorab H, Ng SC, Ng SSM, Zheng S, Chan FKL, Sung JJY, Yu J (2017). Fecal bacteria act as novel biomarkers for noninvasive diagnosis of colorectal cancer. Clin Cancer Res.

[33] Baxter NT, Ruffin MT, Rogers MAM, Schloss PD (2016). Microbiota-based model improves the sensitivity of fecal immunochemical test for detecting colonic lesions. Genome Med.

[34] Zeller G, Tap J, Voigt AY, Sunagawa S, Kultima JR, Costea PI, Amiot A, Böhm J, Brunetti F, Habermann N, Hercog R, Koch M, Luciani A, Mende DR, Schneider MA, Schrotz-King P, Tournigand C, Tran van Nhieu J, Yamada T, Zimmermann J, Benes V, Kloor M, Ulrich CM, Knebel Doeberitz M, Sobhani I, Bork P (2014). Potential of fecal microbiota for early-stage detection of colorectal cancer. Mol Syst Biol.

[35] Ahn J, Sinha R, Pei Z, Dominianni C, Wu J, Shi J, Goedert JJ, Hayes RB, Yang L (2013). Human gut microbiome and risk for colorectal cancer. J Natl Cancer Inst.

[36] Vogtmann E, Hua X, Zeller G, Sunagawa S, Voigt AY, Hercog R (2016). Colorectal cancer and the human gut microbiome: reproducibility with whole-genome shotgun sequencing. PLoS One.

[37] Feng Q, Liang S, Jia H, Stadlmayr A, Tang L, Lan Z, et al. Gut microbiome development along the colorectal adenoma-carcinoma sequence. Nat Commun. 2015;6(1). 10.1038/ncomms7528. 10.1038/ncomms7528 25758642

[38] Yu J, Feng Q, Wong SH, Zhang D, Yi Liang Q, Qin Y (2017). Metagenomic analysis of faecal microbiome as a tool towards targeted non-invasive biomarkers for colorectal cancer. Gut..

[39] Hale VL, Chen J, Johnson S, Harrington SC, Yab TC, Smyrk TC, Nelson H, Boardman LA, Druliner BR, Levin TR, Rex DK, Ahnen DJ, Lance P, Ahlquist DA, Chia N (2017). Shifts in the fecal microbiota associated with adenomatous polyps. Cancer Epidemiol Biomark Prev.

[40] Rothschild D, Weissbrod O, Barkan E, Kurilshikov A, Korem T, Zeevi D, et al. Environment dominates over host genetics in shaping human gut microbiota. Nature Publishing Group; 2018; 10.1038/nature25973 29489753

[41] Song M, Chan AT, Sun J (2020). Influence of the gut microbiome, diet, and environment on risk of colorectal Cancer. Gastroenterology..

[42] Song M, Chan AT. Environmental Factors, Gut Microbiota, and Colorectal Cancer Prevention. Clinical Gastroenterology and Hepatology. Am Gastroenterological Association; 2019. 275–289 p. 10.1016/j.cgh.2018.07.012. . 10.1016/j.cgh.2018.07.012 PMC6314893 30031175

[43] Scott AJ, Alexander JL, Merrifield CA, Cunningham D, Jobin C, Brown R, Alverdy J, O’Keefe SJ, Gaskins HR, Teare J, Yu J, Hughes DJ, Verstraelen H, Burton J, O’Toole PW, Rosenberg DW, Marchesi JR, Kinross JM (2019). International Cancer microbiome consortium consensus statement on the role of the human microbiome in carcinogenesis. Gut..

[44] Thomas AM, Manghi P, Asnicar F, Pasolli E, Armanini F, Zolfo M, Beghini F, Manara S, Karcher N, Pozzi C, Gandini S, Serrano D, Tarallo S, Francavilla A, Gallo G, Trompetto M, Ferrero G, Mizutani S, Shiroma H, Shiba S, Shibata T, Yachida S, Yamada T, Wirbel J, Schrotz-King P, Ulrich CM, Brenner H, Arumugam M, Bork P, Zeller G, Cordero F, Dias-Neto E, Setubal JC, Tett A, Pardini B, Rescigno M, Waldron L, Naccarati A, Segata N (2019). Metagenomic analysis of colorectal cancer datasets identifies cross-cohort microbial diagnostic signatures and a link with choline degradation. Nat Med.

[45] Wirbel J, Pyl PT, Kartal E, Zych K, Kashani A, Milanese A, Fleck JS, Voigt AY, Palleja A, Ponnudurai R, Sunagawa S, Coelho LP, Schrotz-King P, Vogtmann E, Habermann N, Niméus E, Thomas AM, Manghi P, Gandini S, Serrano D, Mizutani S, Shiroma H, Shiba S, Shibata T, Yachida S, Yamada T, Waldron L, Naccarati A, Segata N, Sinha R, Ulrich CM, Brenner H, Arumugam M, Bork P, Zeller G (2019). Meta-analysis of fecal metagenomes reveals global microbial signatures that are specific for colorectal cancer. Nat Med.

[46] Randel KR, Schult AL, Botteri E, Hoff G, Bretthauer M, Ursin G (2020). Colorectal cancer screening with repeated fecal immunochemical test versus sigmoidoscopy: baseline results from a randomized trial.

[47] Norwegian Institute of Public Health (NIPH). Norwegian Prescription Database (NorPD). https://www.fhi.no/en/hn/health-registries/norpd/. Accessed 5 November 2020.

[48] Cancer Registry of Norway. Cancer in Norway 2019 - Cancer incidence, mortality, survival and prevalence in Norway. Oslo; 2020.

[49] Vandenbroucke JP, Von Elm E, Altman DG, Gøtzsche PC, Mulrow CD, Pocock SJ (2007). Strengthening the reporting of observational studies in epidemiology (STROBE): explanation and elaboration. PLoS Med.

[50] Vandenbroucke JP, von Elm E, Altman DG, Gøtzsche PC, Mulrow CD, Pocock SJ, Poole C, Schlesselman JJ, Egger M, STROBE Initiative (2014). Strengthening the reporting of observational studies in epidemiology (STROBE): explanation and elaboration. Int J Surg.

[51] Bharucha T, Oeser C, Balloux F, Brown JR, Carbo EC, Charlett A, Chiu CY, Claas ECJ, de Goffau MC, de Vries JJC, Eloit M, Hopkins S, Huggett JF, MacCannell D, Morfopoulou S, Nath A, O'Sullivan DM, Reoma LB, Shaw LP, Sidorov I, Simner PJ, van Tan L, Thomson EC, van Dorp L, Wilson MR, Breuer J, Field N (2020). STROBE-metagenomics: a STROBE extension statement to guide the reporting of metagenomics studies. Lancet Infect Dis.

[52] Hassan C, Quintero E, Dumonceau JM, Regula J, Brandão C, Chaussade S, Dekker E, Dinis-Ribeiro M, Ferlitsch M, Gimeno-García A, Hazewinkel Y, Jover R, Kalager M, Loberg M, Pox C, Rembacken B, Lieberman D, European Society of Gastrointestinal Endoscopy (2013). Post-polypectomy colonoscopy surveillance: European Society of Gastrointestinal Endoscopy (ESGE) guideline. Endoscopy..

[53] Rounge TB, Meisal R, Nordby JI, Ambur OH, De Lange T, Hoff G (2018). Evaluating gut microbiota profiles from archived fecal samples. BMC Gastroenterol.

[54] University of Oslo (UiO). About TSD. https://www.uio.no/english/services/it/research/sensitive-data/about/index.html. Accessed 14 December 2020.

[55] Köster J, Rahmann S (2018). Snakemake - a scalable bioinformatics workflow engine. Bioinformatics..

[56] Bolger AM, Lohse M, Usadel B (2014). Trimmomatic: a flexible trimmer for Illumina sequence data. Bioinformatics..

[57] Langmead B, Salzberg SL (2012). Fast gapped-read alignment with bowtie 2. Nat Methods.

[58] Ewels P, Magnusson M, Lundin S, Käller M (2016). MultiQC: summarize analysis results for multiple tools and samples in a single report. Bioinformatics..

[59] Andersen LF, Solvoll K, Johansson LRK, Salminen I, Aro A, Drevon CA (1999). Evaluation of a food frequency questionnaire with weighed records, fatty acids, and alpha-tocopherol in adipose tissue and serum. Am J Epidemiol.

[60] Andersen LF, Tomten H, Haggarty P, Løvø A, Hustvedt BE (2003). Validation of energy intake estimated from a food frequency questionnaire: a doubly labelled water study. Eur J Clin Nutr.

[61] Carlsen MH, Lillegaard IT, Karlsen A, Blomhoff R, Drevon CA, Andersen LF (2010). Evaluation of energy and dietary intake estimates from a food frequency questionnaire using independent energy expenditure measurement and weighed food records. Nutr J.

[62] Andersen LF, Veierød MB, Johansson L, Sakhi A, Solvoll K, Drevon CA (2005). Evaluation of three dietary assessment methods and serum biomarkers as measures of fruit and vegetable intake, using the method of triads. Br J Nutr.

[63] Carlsen MH, Karlsen A, Lillegaard ITL, Gran JM, Drevon CA, Blomhoff R, Andersen LF (2011). Relative validity of fruit and vegetable intake estimated from an FFQ, using carotenoid and flavonoid biomarkers and the method of triads. Br J Nutr.

[64] Brunvoll SH, Thune I, Frydenberg H, Flote VG, Bertheussen GF, Schlichting E (2018). Validation of repeated self-reported n-3 PUFA intake using serum phospholipid fatty acids as a biomarker in breast cancer patients during treatment. Nutr J.

[65] Norwegian Food Safety Authority. Norwegian Food Composition Database 2019 [Internet]. Available from: www.matvaretabellen.no. .

[66] Johansson I, Hallmans G, Wikman Å, Biessy C, Riboli E, Kaaks R (2002). Validation and calibration of food-frequency questionnaire measurements in the northern Sweden health and disease cohort. Public Health Nutr.

[67] Holmberg L, Ohlander EM, Byers T, Zack M, Wolk A, Bruce Å, Bergstrom R, Bergkvist L, Adami HO (1996). A search for recall Bias in a case-control study of diet and breast Cancer. Int J Epidemiol.

[68] Willett W (2013). Nutritional epidemiology. Oxford.

[69] World Cancer Research Fund/American Institute for Cancer Research. Diet, nutrition, physical activity and colorectal cancer. Continuous Update Project. 2018. https://www.wcrf.org/sites/default/files/Colorectal-cancer-report.pdf%0Ahttps://www.wcrf.org/sites/default/files/Oesophageal-cancer-report.pdf. Accessed 8 June 2021.

[70] Shams-White MM, Brockton NT, Mitrou P, Romaguera D, Brown S, Bender A, Kahle LL, Reedy J (2019). Operationalizing the 2018 World Cancer Research Fund/American Institute for Cancer Research (WCRF/AICR) Cancer prevention recommendations: a standardized scoring system. Nutrients..

[71] Markussen MS, Veierod MB, Kristiansen AL, Ursin G, Andersen LF (2016). Dietary patterns of women aged 50-69 years and associations with nutrient intake, sociodemographic factors and key risk factors for non-communicable diseases. Public Health Nutr.

[72] Knudsen MD, Berstad P, Hjartåker A, Gulichsen EH, Hoff G, De Lange T (2017). Lifestyle predictors for non-participation and outcome in the second round of faecal immunochemical test in colorectal cancer screening. Br J Cancer.

[73] Folkehelseinstituttet. Reseptregisteret 2012-2016. 2017.

[74] WHO Collaborating Centre for Drug Statistics Methodology. Structure and principles https://www.whocc.no/atc/structure_and_principles/. Accessed 28 September 2020.

[75] WHO Collaborating Centre for Drug Statistics Methodology. ATC/DDD Index 2020 https://www.whocc.no/atc_ddd_index/. Accessed 28 September 2020.

[76] Cancer Registry of Norway. Cancer in Norway 2018 - Cancer incidence, mortality, survival and prevalence in Norway. Oslo; 2019.

[77] Larsen IK, Smastuen M, Johannesen TB, Langmark F, Parkin DM, Bray F (2009). Data quality at the Cancer registry of Norway: an overview of comparability, completeness, validity and timeliness. Eur J Cancer England.

[78] Norwegian Institute of Public Health (NIPH). Access to data from the Norwegian Prescription Database. https://www.fhi.no/en/hn/health-registries/norpd/Access-data-norpd/#legal-requirements-for-the-disclosure-of-data-from-the-norpd. Accessed 19 June 2020.

[79] Cancer Registry of Norway. The microbiome as a colorectal cancer screening biomarker. https://www.kreftregisteret.no/en/Research/Projects/microbiota-and-lifestyle-in-colorectal-cancer-screeing/. Accessed 29 September 2020.

[80] Tibshirani R (1996). Regression shrinkage and selection via the lasso. J R Stat Soc Ser B.

[81] Cortes C, Vapnik V. Support-vector networks. Mach Learn. 1995;

[82] Breiman L. Random forests. Mach Learn. 2001:1–122.

[83] Haykin S. Neural networks and learning machines. Third Edit. Pearson Prentice Hall; 2009.

[84] Chen T, Guestrin C. XGBoost: A Scalable Tree Boosting System. Proc 22nd ACM SIGKDD Int Conf Knowl Discov Data Min. 2016;

[85] Stark M, Zapf A. Sample size calculation and re-estimation based on the prevalence in a single-arm confirmatory diagnostic accuracy study. Stat Methods Med Res. 2020; 10.1177/0962280220913588 32299298

[86] Nagata N, Tohya M, Fukuda S, Suda W, Nishijima S, Takeuchi F, Ohsugi M, Tsujimoto T, Nakamura T, Shimomura A (2019). Effects of bowel preparation on the human gut microbiome and metabolome. Sci Rep.

[87] Ahlquist DA, McGill DB, Fleming JL, Schwartz S, Wieand HS, Rubin J, Moertel CG (1989). Patterns of occult bleeding in asymptomatic colorectal cancer. Cancer..

[88] Hannelore D. Diet and the gut microbiome: from hype to hypothesis. Br J Nutr. 2020:1–24. 10.1017/S0007114520001142 32238220

[89] Berstad P, Løberg M, Larsen IK, Kalager M, Holme Ø, Botteri E, Bretthauer M, Hoff G (2015). Long-term lifestyle changes after colorectal cancer screening: randomised controlled trial. Gut..

[90] Knudsen MD, Hjartåker A, Olsen MKE, Hoff G, De Lange T, Bernklev T (2018). Changes in health behavior 1 year after testing negative at a colorectal cancer screening: a randomized-controlled study. Eur J Cancer Prev.

