# Supplementary material for: The CRCbiome study: a large prospective cohort study examining the role of lifestyle and the gut microbiome in colorectal cancer screening participants
Source: BMC Cancer. 2021 Aug 18;21:930. doi: 10.1186/s12885-021-08640-8 (PMC8371800; doi:10.1186/s12885-021-08640-8)
Supplement: Supplementary file 2 — Additional file 2. Contains a supplementary figure with figure title and legend. [file 12885_2021_8640_MOESM2_ESM.docx]

**Additional file 2: Supplementary Figures**

**
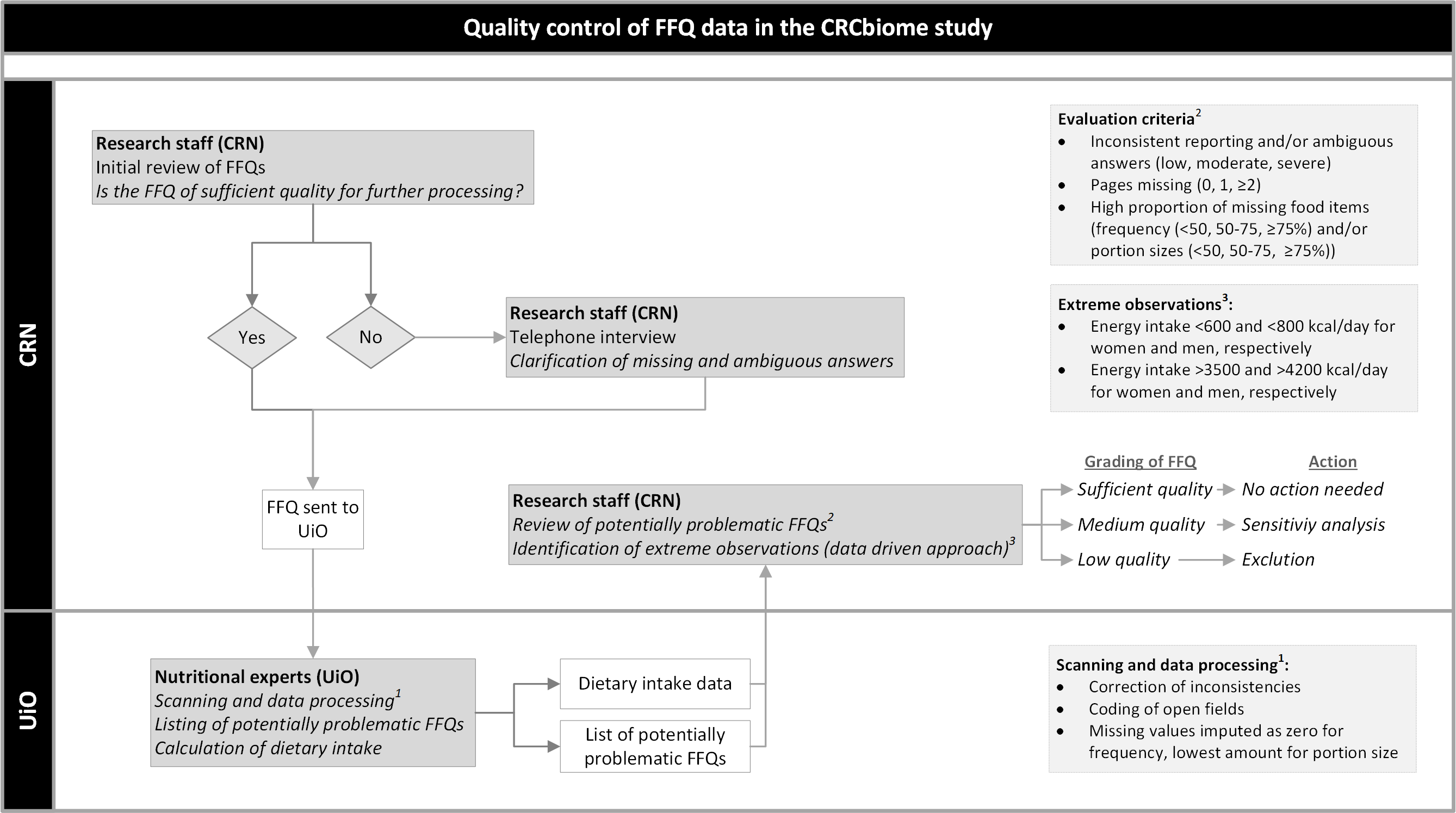
**

**Figure 1.** Upon receiving food frequency questionnaires (FFQs) from CRCbiome participants, completion is reviewed by researchers with expertise in nutritional epidemiology. Participants with FFQs of insufficient quality are contacted for clarification of inconsistencies and missing data. Reviewed questionnaires are then scanned using the Cardiff TeleForm program at the University of Oslo (UiO). Food and nutrient calculations are conducted using the software system KBS (“**K**ost**b**eregnings**s**ystem”/Dietary Calculation System) with the latest version of the food database, largely based on the Norwegian Food Composition Table (1). Missing answers are imputed as zero in line with common practice (2–5). Any FFQs regarded as potentially problematic during the data handling process are listed. Dietary intake data and the list of potentially problematic FFQs are then returned to the Cancer Registry of Norway (CRN). Potentially problematic FFQs are reviewed according to a set of predefined criteria, including inconsistency in reporting, number of missing pages and amount of missing food items. Based on these criteria, FFQs are graded as being of low, medium or sufficient quality. Whereas low quality FFQs will be excluded from all analysis where diet is the primary exposure, medium quality FFQs will be included unless sensitivity analysis indicates substantial attenuation of effect estimates. Lastly, in line with common practice in nutrition studies (6), observations with extreme energy intake levels in both the upper and lower range will be excluded.

**References**

1. Norwegian Food Safety Authority. Norwegian Food Composition Database 2019. [cited 2020 Jun 16]. http://www.matvaretabellen.no

2. Johansson I, Hallmans G, Wikman Å, Biessy C, Riboli E, Kaaks R. Validation and calibration of food-frequency questionnaire measurements in the Northern Sweden Health and Disease cohort. Public Health Nutr. 2002;5:487–96.

3. Carlsen MH, Lillegaard IT, Karlsen A, Blomhoff R, Drevon CA, Andersen LF. Evaluation of energy and dietary intake estimates from a food frequency questionnaire using independent energy expenditure measurement and weighed food records. Nutr J. 2010;9:1–9.

4. Carlsen MH, Karlsen A, Lillegaard ITL, Gran JM, Drevon CA, Blomhoff R, Andersen LF. Relative validity of fruit and vegetable intake estimated from an FFQ, using carotenoid and flavonoid biomarkers and the method of triads. Br J Nutr. 2011;105:1530–8.

5. Holmberg L, Ohlander EM, Byers T, Zack M, Wolk A, Bruce Å, Bergstrom R, Bergkvist L, Adami HO. A Search for Recall Bias in a Case-Control Study of Diet and Breast Cancer. Int J Epidemiol. 1996;25:235–44.

6. Willett W. Nutritional epidemiology. Oxford; New York: Oxford University Press; 2013.
